# A two-tier strategy for developing water deficit stress tolerant maize: hydroponics-based root phenotyping followed by rainfed field validation

**DOI:** 10.3389/fpls.2025.1716676

**Published:** 2026-02-24

**Authors:** Rutuparna Pati, Surinder Sandhu, Yogesh Vikal, Manje Gowda, P. H. Zaidi, Rumesh Ranjan, M. T. Vinayan, B. S. Vivek, Rakesh Sharda, Tosh Garg

**Affiliations:** 1 Department of Plant Breeding and Genetics, Punjab Agricultural University, Ludhiana, Punjab, India; 2 India School of Agricultural Biotechnology, Punjab Agricultural University, Ludhiana, Punjab, India; 3 International Maize and Wheat Improvement Centre (CIMMYT), Nairobi, Kenya; 4 The International Maize and Wheat Improvement Centre (CIMMYT)-Hyderabad, Patancheru, India; 5 Department of Soil and Water Engineering, Punjab Agricultural University, Ludhiana, Punjab, India

**Keywords:** ASI, maize, water deficit stress, hydroponics, climate resilience

## Abstract

Maize productivity is increasingly constrained by water deficit stress (WDS), particularly under erratic rainfall conditions. Efficient early-stage phenotyping coupled with field validation is critical for breeding WDS-tolerant genotypes. In this study, we developed a two-tier screening strategy integrating hydroponics-based root trait evaluation at pre-reproductive stage with subsequent field validation of maize inbreds under managed WDS at CIMMYT, Hyderabad. A set of 50 diverse maize inbreds were evaluated for root architectural traits and plant growth stages including grain yield components. Hydroponic screening applied PEG6000-induced osmotic stress to assess root length, tips, forks, segments and diameter, whereas field trials imposed pre-reproductive WDS through cumulative growing degree day-based irrigation withdrawal. Significant genotypic variation and genotype × trait interactions were observed across both environments, reflecting trait and environment-specific responses. Key root traits, including root tips, total length, forks and segments, showed strong positive correlations (r ≥ 0.70) with yield components and Normalized difference vegetation index (NDVI), underscoring their importance in WDS resilience. Multivariate analysis further confirmed the alignment of root vigor with kernel traits and canopy health as critical determinants of yield stability. Among the evaluated lines, introgressed ILM23 and ILM24 emerged as the principal donor lines, while PML1249, PML1275, and PML1285 were identified as promising donor sources, all exhibiting robust root systems, stable anthesis-silking interval (ASI) and superior stress tolerance indices. Spearman’s rank correlation (ρ = 0.988) between hydroponics and field rankings highlighted the predictive reliability of controlled root phenotyping for field performance under WDS. This integrated hydroponics-to-field approach provides a rapid, efficient and cost-effective framework for the early identification of WDS-tolerant or high water-use-efficiency (WUE) maize hybrids, facilitating the accelerated breeding of resilient cultivars.

## Introduction

Maize, one of the prominent crops, is frequently exposed to various biotic and abiotic stresses that pose significant challenges in maintaining overall crop productivity (Sah et al., 2020). Among the many unprecedented challenges, water deficit and erratic rainfall patterns pose major threats to global crop production. Erratic rainfall patterns during the early growth stages disrupt soil moisture availability, thereby exposing crops to water deficit stress (WDS) from the pre-flowering stage through to late grain filling (Sathish et al., 2022). These challenges significantly affect the morphology, flowering behavior and grain development of the crop. Consequently, maize production during the wet season and in rain-fed regions is declining due to natural, intermittent WDS (Kaur et al., 2025). With ongoing climatic changes, it is imperative to focus on maintaining maize yields by identifying genotypes that are tolerant to WDS and promoting breeding techniques and management methods that can protect maize from WDS in the future. This necessitates the rethinking and redesigning of breeding strategies, specifically to target key component traits to identify genotypes with higher productivity and better tolerance to stresses posed by the changing climate. In addition, an upgraded understanding of the complex correlations among morphological features linked to WDS tolerance has rational implications for recognizing several tolerance mechanisms that will assist in ameliorating the adverse effects of WDS on maize. In WDS, crops preferentially provide limited water to the root system, which is an important organ for crops to supplement nutrients and absorb water (Sathish et al., 2022). Plants can first sense changes in the soil environment and then take the initiative to find places with sufficient soil moisture content, mainly by elongating and increasing fine roots to improve the water absorption capacity of the roots and ensure crop growth and development (Wajhat-un-nisa et al., 2023). Root system architecture plays a central role in determining plant water status during critical growth stages and thereby directly influences yield components such as anthesis–silking interval, kernel set (Deribe, 2025). A deeper understanding of the relationship between root traits and yield-related parameters is therefore essential for effective selection of WDS-tolerant maize genotypes. However, characterizing roots in both field and pot experiments is a complex task and there is currently no precise and user-friendly method for assessing roots under abiotic stress in these experiments (Trachsel et al., 2013). With the ongoing problem, hydroponic systems can serve as a rapid and efficient early screening technique to assess a wide range of major crop traits during the seedling stage. Hydroponics frequently employs non-ionic osmotica to address heterogeneity, drainage, and fluctuations in water potential. Hence, the low-volume hydroponic method can be readily adapted for secretion sampling, root-labeling experiments, and continuous or pulse-type root-feeding studies in maize and other large C_4_ grasses, such as sorghum, sugarcane, and miscanthus (Ghazi, 2021). Here, we developed a two-tier strategy for WDS screening in maize: (i) hydroponics-based phenotyping of root architectural traits at pre flowering stage with osmotic agents (PEG/non-ionic) and (ii) validation of these traits under a managed water deficit in the field. This integrated approach will enable the early identification of genotypes with robust root systems and high WUE, supporting the accelerated breeding of WDS-tolerant maize hybrids.

## Materials and methods

A set of 50 maize inbred lines of diverse origin, comprising LM/PML lines developed by Punjab Agricultural University, Ludhiana, and VL series sourced from CIMMYT Asia, Hyderabad, were used for the present study. In addition, two introgressed lines, ILM23 and ILM24 (prefix I denotes introgressed), developed via marker-assisted breeding to introgress major WDS-tolerance QTL (Kaur et al., 2025) and their untransformed counterparts LM 23 and LM 24 were also included in the study. Test lines are being maintained through selfing/sib-mating.

### Experiment 1: hydroponic-based early-stage screening of maize inbreds

#### Growth conditions and experimental design

Maize inbreds were screened for WDS tolerance under a polyhouse hydroponic system during 2023–24. Seeds were surface sterilized, germinated in coco-coir for 15 days, and transferred to meshed pots containing clay balls. Seedlings under optimal water conditions served as controls, whereas WDS was induced at V_5_ stage for a period of 15 days (pre-flowering to flowering) using 10% PEG-6000 in the optimized Hoagland’s nutrient solution (**Table 1**). The nutrient solution was replaced every 3–5 days and the pH was monitored daily and maintained at 7.0 ± 0.2. Solutions were intermittently aerated to maintain sufficient oxygen supply to the roots. The experiment followed a randomized complete block design with two replications per treatment with each replication consisted of 3 plants per genotype for both control and WDS treatments. Polyhouse conditions were maintained at 16 h light/8 h dark, 25 °C/18 °C (day/night) and 65% relative humidity. Chlorophyll was measured at five leaf stages using an MC-100 meter. At 60 DAS, the roots were harvested and scanned using built-in software Biovis PSM-R2000 to determine the projection area, diameter, volume, tips, forks, and total length.

**Table 1:** Protocol optimization for hydroponic-based phenotyping (list of macro- and micro-nutrient concentrations standardized and used for growing maize seedlings in a hydroponic medium).

Chemicals	Mol wt.	gm/100 lit
Macronutrients		
Calcium nitrate (Ca(NO_3_)_2_)	236.1	24.6
Potassium nitrate (KNO_3_)	101.1	15.1
Ammonium dihydrogen phosphate (NH_4_H_2_PO_4_)	115.1	11.5
Magnesium sulfate heptahydrate (MgSO_4_·7H_2_O)	246.5	54.6
Potassium dihydrogen phosphate (KH_2_PO_4_)	136.1	2.5
Micronutrients		
Potassium chloride (KCl)	74.55	0.186
Boric acid (H_3_BO_3_)	61.83	0.077
Manganese (II) sulfate monohydrate (MnSO_4_·H_2_O)	169	0.017
Zinc sulfate heptahydrate (ZnSO_4_·7H_2_O)	287.5	0.029
Copper (II) sulfate pentahydrate (CuSO_4_·5H_2_O)	249.7	0.36
Sodium molybdate (Na_2_MoO_3_)	244.1	0.002
Sodium ferric ethylenediaminetetraacetate (Na-Fe EDTA)	558.5	3.293
WDS treatment		
Polyethylene glycol-6000 (PEG-6000)		10Kg

### Experiment 2: field validation under managed WDS

**Figure 1:**
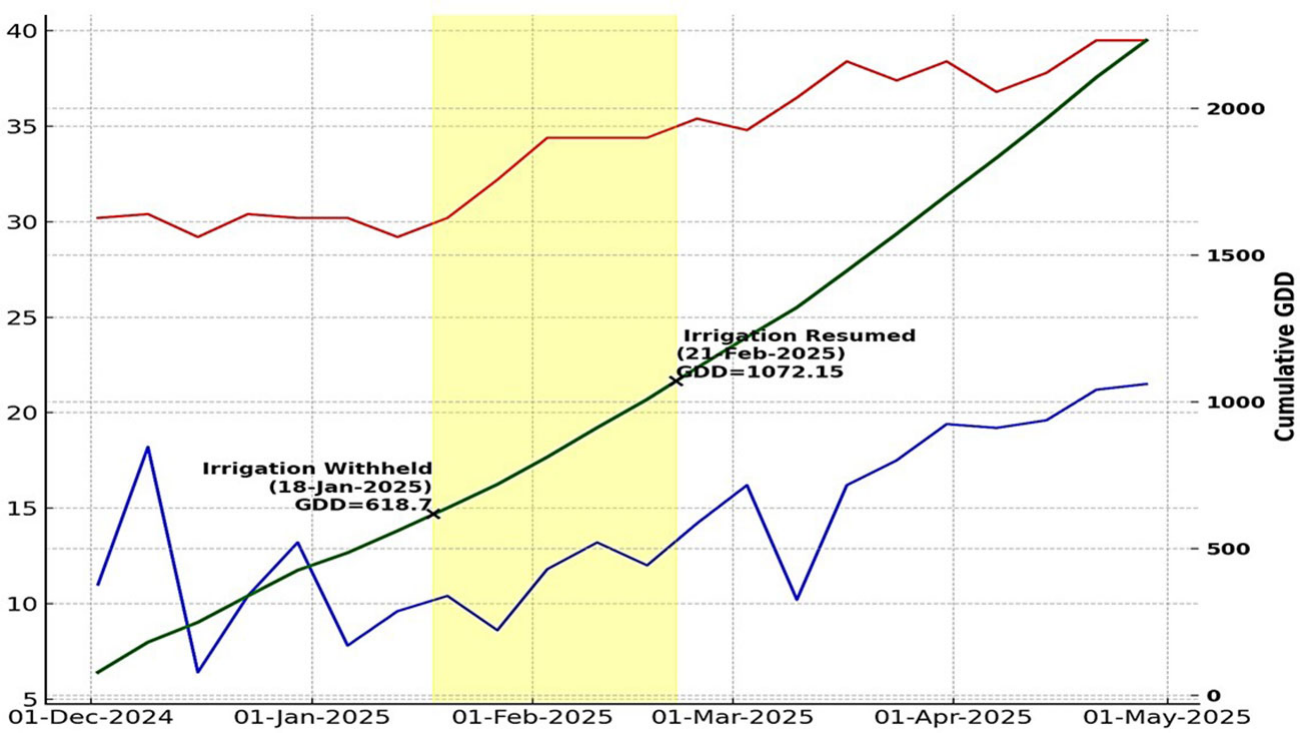
Weekly minimum and maximum temperature, Cumulative Growing Degree Days (GDD), and WDS period.


A field evaluation trial was conducted at the CIMMYT–ICRISAT campus, Patancheru, Hyderabad, India (17.5036° N, 78.2789° E; 545 m above mean sea level) during the *rabi* season of 2024–25 on sandy loam soil. This field location in the Deccan Plateau is prone to intermittent water deficits during the period. The trial was laid out in a randomized block design in two replications, with each genotype in a plot size of 4.5 square meters, in two rows of 3m each at row-to-row and plant-to-plant spacing of 75 × 20 cm, respectively. The trial was sown on November 24, 2024, and harvested on April 26, 2025. All recommended agronomic and cultural operations were followed to raise the crop. No rainfall was recorded during the trial duration. Pre-reproductive WDS was imposed by withholding irrigation according to cumulative growing degree days (GDD), from 618.7 to 1,072.15 GDD, followed by “rescue irrigation” to prevent irreversible wilting, that is, 16.8% v/v at a soil depth of 30–40 cm (**Figure 1**). Soil moisture was monitored at 10-cm intervals up to 100 cm using a Delta-T PR2 soil moisture probe to maintain a consistent stress intensity. Water deficit protocols by CIMMYT were strictly followed during the managed stress trials (Zaman-Allah et al., 2016; Zaidi et al., 2016). Cumulative growing degree days (GDD) were calculated from the day irrigation was withheld (January 18, 2025) until irrigation was resumed (February 21, 2025) to ensure accurate stress intensity at the target stage of the crop.

$$GDD=\left(T_{max}+T_{min}\right)/2)–T_{base}$$

where:

T_max_ = daily maximum temperature
T_min_​ = daily minimum temperature
T_base_ base temperature (crop-specific, usually the minimum threshold for growth, e.g., 10 °C for maize (Gilmore and Rogers, 1958).

### Data collection and statistical analysis

Fifteen phenotypic traits were recorded, including flowering, vegetative, ear, and yield attributes. Flowering traits included days to 50% anthesis (DA), recorded as the number of days from sowing until 50% of plants extruded anthers, and the anthesis–silking interval (ASI) at 50%, calculated as the difference in days between 50% anthesis and 50% silking to assess the synchrony of male and female flowering. Vegetative and leaf traits included plant height (PH, cm), number of leaves per plant (NL), SPAD chlorophyll index, Normalized difference vegetation index(NDVI), leaf length (LL, cm), leaf width (LW, cm), and number of plants per plot (NP). Ear traits included ear height (EH, cm) and ear length (EL, cm), whereas yield traits included the number of kernels per row (NR), number of kernel rows per ear (NKR), cob length (CL, cm), cob breadth (CB, mm), and grain yield per row (kg/plot). All observations were recorded following standard procedures to ensure accuracy and reliability, with flowering traits such as ASI particularly useful for evaluating WDS tolerance and stress response, whereas vegetative and ear traits provided information on plant vigor and yield potential under field conditions. The root traits of individual inbred under control (Y_N_) and WDS (Y_H_) conditions, along with the mean trait data of all inbred lines in respective environments (X_N_ and X_H_), were utilized to compute stress tolerance indices.

The following calculations were used to compute the WDS tolerance indices:

Stress tolerance index (STI) = (Y_N_ × Y_S_)/X_S_^2^ (Fernandez, 1992)
Stress tolerance (TOL) = Y_N_ – Y_S_ (Rosielle and Hamblin, 1981)

The collected data were statistically analyzed using R version 4.5.0.

**Figure 2:**
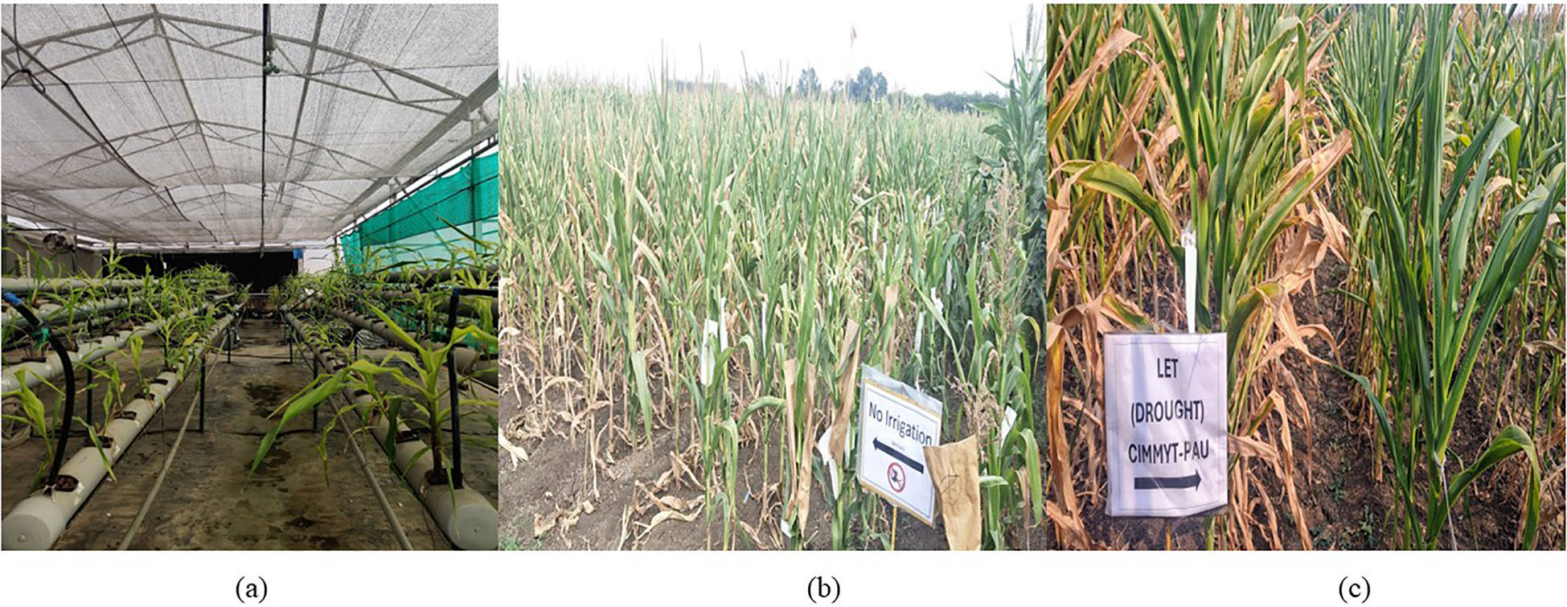
Screening of maize inbred lines under water deficit stress conditions. **(a)** Hydroponic platform for early-stage root trait evaluation at 30 DAS. **(b, c)** Field evaluation under managed drought stress at CIMMYT, Hyderabad (no irrigation imposed at vegetative to flowering stage).

The two-tier screening is depicted in **Figure 2**.

## Results

### Optimization of hydroponic-based phenotyping protocol

The hydroponic protocol was successfully standardized for early-stage phenotyping of maize under WDS (**Table 1**). Macronutrients and micronutrients were optimized to ensure balanced seedling growth, while a 10% PEG-6000 solution was incorporated 45 days after sowing to simulate osmotic stress without ionic toxicity, providing a controlled environment for evaluating root and shoot responses. This optimized nutrient composition and stress application allowed consistent and reproducible growth of maize seedlings in the hydroponic medium, supporting accurate phenotyping of root architectural traits and early stress responses. Overall, the protocol provides a cost-effective, rapid, and scalable method for screening large numbers of maize genotypes for WDS tolerance, establishing a reliable foundation for subsequent correlation with field performance under WDS conditions.

### Analysis of variance for root and yield traits

**Figure 3:**
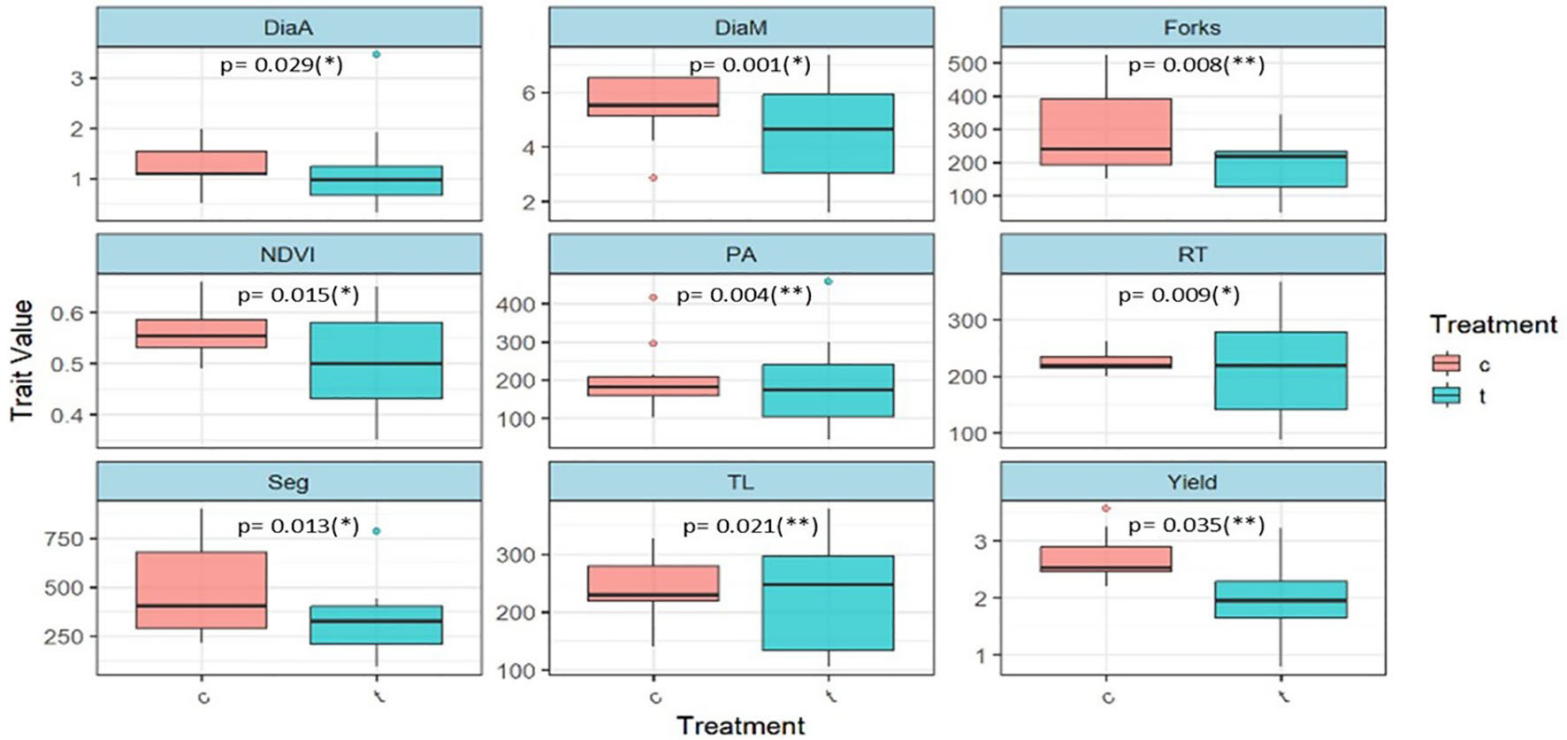
Boxplots showing variation in traits under control (c) and water-deficit stress (t) treatments. Statistical significance between treatments was evaluated using independent t-tests, and the corresponding p-values with significance levels (*p < 0.05; **p<0.01; ***p < 0.001) are indicated within each panel. Lower p-values reflect stronger treatment effects on the respective traits.

The ANOVA for root traits under the hydroponics platform (**Table 2**) revealed highly significant differences among genotypes (p< 0.001), traits (p< 0.001), and genotype × trait interaction (p< 0.001), indicating substantial genetic variability and trait-specific effects. Water deficit treatment showed a significant effect (p< 0.01), whereas replication was non-significant, suggesting minimal experimental error. Similarly, field evaluation for yield and its contributing traits (**Table 3**) demonstrated highly significant variation among genotypes (p< 0.001), traits (p< 0.001), and genotype × trait interactions (p< 0.001). Replication effects were non-significant, confirming the uniform trial conditions. These results highlight the presence of strong genotypic differences and trait-specific responses, supporting the effectiveness of both hydroponic and field platforms in identifying WDS-tolerant maize genotypes. Boxplots of root traits (**Figure 3**) illustrated substantial variation among inbreds under WDS. Traits such as DiaM, Forks and Seg were reduced under stress, RT and TL increased, suggesting adaptive or compensatory root responses. NDVI and PA showed moderate reductions under WDS, with notable genotypic variation.

**Table 2:** Analysis of variance of maize stock for root traits under hydroponics phenotyping platform.

Source	Sum square	Mean Sq	F Value	Pr(>F)
Gen	8,95,577	99,509	17.88	<2e-16 ***
Trait	76,68,163	9,58,520	172.24	<2e-16 ***
Treatment	36,585	36,585	6.57	0.0109 *
Rep	14,824	14,824	2.66	0.1038 ns
Gen × Trait	18,05,651	25,078	4.51	<2e-16 ***
Residuals	14,91,467	5,565		

***: p<0.001 (Highly Significant) ∗: p<0.01 (Significant), ns: p≥0.05 (non-significant).

**Table 3:** Analysis of variance of maize stock for yield and its contributing traits under field conditions.

Source	Sum square	Mean Sq	F Value	Pr(>F)
Gen	6,504	186	21.27	<2e-16 ***
Trait	12,17,968	86,998	9,957.65	<2e-16 ***
Rep	1	1	0.132	0.717ns
Gen × Trait	55,252	113	12.91	<2e-16 ***
Residuals	4,447	9		

***: p<0.001 (Highly Significant) ∗: p<0.05 (Significant), ns: p≥0.05 (non-significant).

### Root traits associated with physiological, kernel and yield parameters

**Figure 4:**
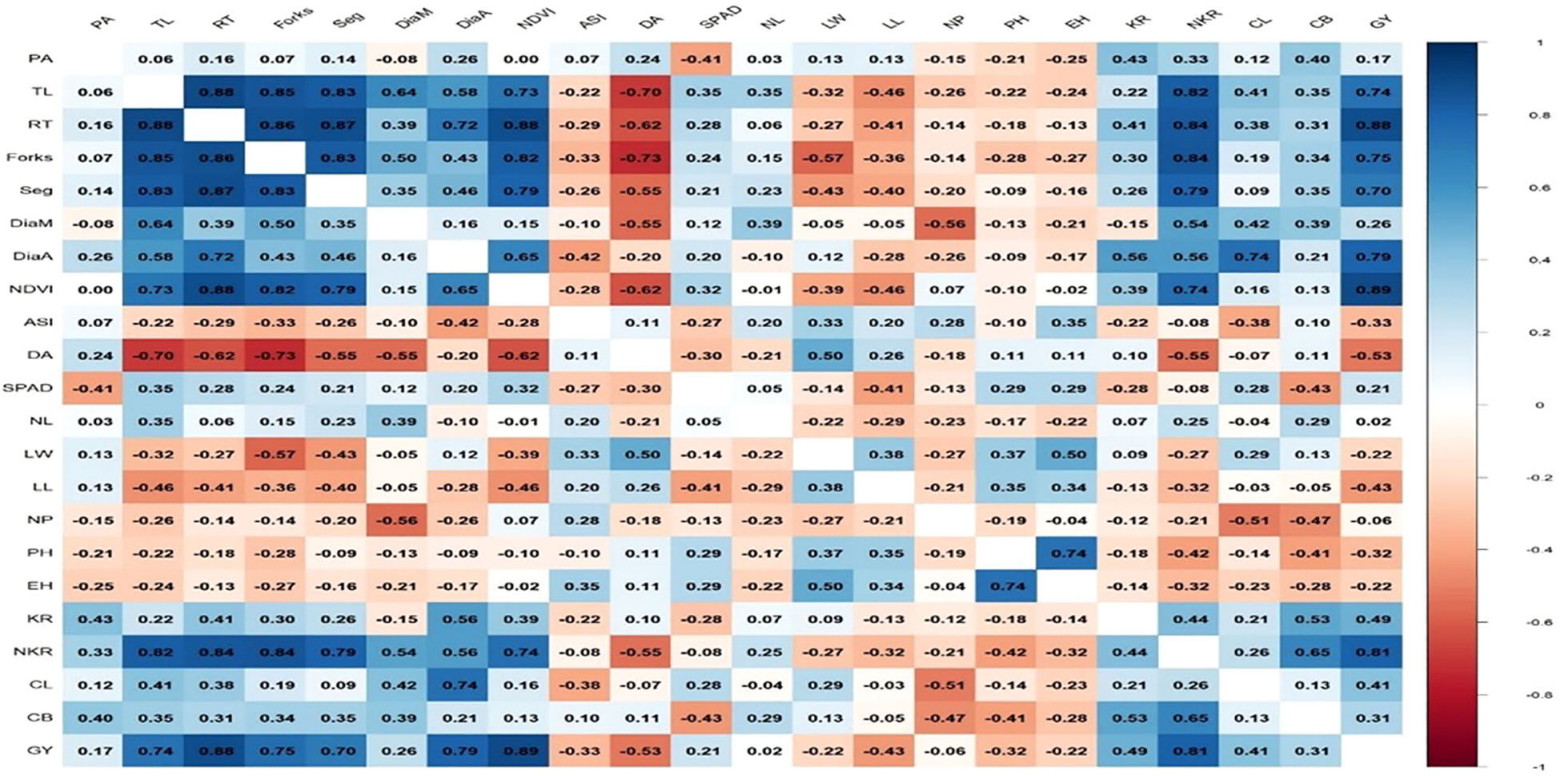
Correlation matrix of maize traits: a visual representation of the linear relationships (R-values) between all measured root, vegetative and yield components. Colour intensity and shade (blue for positive, red for negative) indicate the strength and direction of the correlation between each pair of measured traits. ASI, Anthesis silking interval (days); CB, Cob breadth(mm); CL, Cob length(cm); DA, Days to anthesis; DiaA, Average root diameter(mm; DiaM, Maximum root diameter (mm; EH, Ear height (cm); Forks, total number of forks; GY, Grain yield plot(kg); KR, Total number of kernel rows; NDVI, Normalized difference vegetation index; NKR, Total Number of kernels/row; NL, Total number of leaves; NP, Number of plants LL Leaf length(cm); LW, Leaf width (cm); PA, Root projection area(cm³); PH, Plant height(cm); RT, Root tips(numbers); Seg, Total number of root segments; SPAD, Chlorophyll index; TL, Total Root Length.

To validate the significance of root traits, correlation analysis was performed to highlight the important trait correlations of various yield attributing traits and grain yield. Root traits showed strong positive correlations with yield-attributing traits, confirming their importance in driving the productivity (**Figure 4**). RT exhibited highly significant positive correlations with KR (r = 0.85), NKR (r = 0.84), and GY (r = 0.85), indicating that a robust root system directly supports kernel development and final yield. Similarly, TL correlated positively with NDVI (r = 0.73), KR (r = 0.82), NKR (r = 0.84), and GY (r = 0.74). Other root traits, such as forks (r = 0.82 with KR; r = 0.82 with NKR; r = 0.75 with GY), seg (r = 0.85 with KR; r = 0.87 with NKR; r = 0.75 with GY), and root diameter (DiaA: r = 0.79 with GY; DiaM: r = 0.79 with GY), also showed strong positive associations with yield and its component traits.

### Multivariate analysis of trait associations using principal component analysis

**Figure 5:**
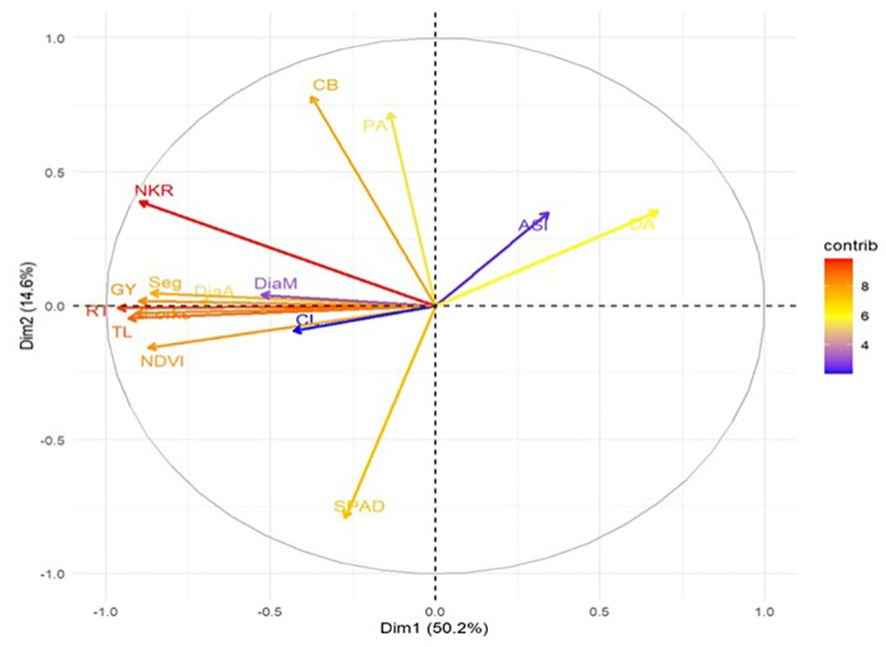
Principal component analysis (PCA) biplot summarizing the variability among maize genotypes (points 1-20) based on hydroponic and field traits (arrows) under WDS. The first two principal components, Dim1 and Dim2, account for 64.8% (50.2% + 14.6%) of the total phenotypic variation. ASI, Anthesis silking interval (days); DiaA, Average root diameter (mm); DiaM, Maximum root diameter (mm); Forks, total number of forks; GY, Grain yield/plot (kg); KR, Total number of kernel rows; NDVI, Normalized difference vegetation index; NKR, Total Number of kernels/row; PA, Root projection area (cm³); RT, Root tips (numbers); Seg, Total number of root segments; SPAD, Chlorophyll index; TL, Total Root Length.

Principal component analysis (PCA) integrated root, physiological, and yield traits to identify the key contributors to WDS tolerance (**Figure 5**). Dim1 explained 50.2% of the variation and was strongly influenced by root traits (RT, Seg, TL, Forks DiaA, DiaM), NDVI, yield attributing traits (KR, NKR, CL, CB), and GY, all of which clustered closely in the positive direction, forming acute angles, indicating their strong interdependence in driving yield performance. Dim2 accounted for 14.6% of the variation and was largely defined by DA, LW, and LL on the positive axis, whereas PH, EH, and NP contributed to the negative axis. ASI, being positioned away from yield and root-related traits, clustered with LL and LW, reflecting its negative relationship with yield and reproductive efficiency. The strong alignment of root traits (RT, TL, Seg, Forks) with NDVI, kernel traits, and yield further validates their role as indirect selection criteria for maintaining greenness and overall productivity of the crop under WDS conditions.

PCA and correlation analyses identified the top traits driving grain yield (GY) under WDS (**Table 4**). Root architectural traits, including Root Tips (RT, r = 0.88**), Total Root Length (TL, r = 0.74**), forks (r = 0.75**) and Root Segments (Seg, r = 0.70**), showed strong positive associations with GY, highlighting the critical role of below-ground resilience in maintaining yield. Among the above-ground traits, NDVI (r = 0.69**) and number of kernels per row (NKR, r = 0.81**) were also strongly correlated with yield, indicating that canopy health and reproductive efficiency complement root performance. Other ear traits, such as cob length (CL, r = 0.41*), cob breadth (CB, r = 0.31*), and kernel rows (KR, r = 0.42*), showed moderate positive associations. Overall, these results confirm that robust root systems combined with efficient aboveground traits determine yield stability under WDS. Top root traits influencing grain yield under WDS (r ≥ 0.70) included RT (r = 0.88), Forks (r = 0.75), TL (r = 0.74), Seg (r = 0.70), NKR (r = 0.81), and NDVI (r = 0.69), reinforcing the importance of below-ground resilience combined with above-ground efficiency (**Table 4**).

**Table 4:** Top contributing root traits to grain yield (GY) under stress: PCA contribution and correlation analysis.

Rank	Trait	Correlation with GY	Interpretation	References
1	RT (Root Tips)	0.88**	Thick roots improve anchorage & water uptake → higher yield.	Sandhu et al., 2025; Kaur et al., 2025; Sah et al., 2020
2	TL (Total Root Length)	0.74**	Longer roots allow deeper water/nutrient capture.	Yetgin, 2024; Sandhu et al., 2025; Ranjan et al., 2022
3	Forks (Root branching)	0.75**	More branching improves soil exploration under stress.	Ranjan et al., 2022; Henry et al., 2011
4	Seg (Root segments)	0.70**	Denser root network supports yield stability.	Sandhu et al., 2025; Asadullah et al., 2024

**p ≤ 0.01.

### Identification of promising genotypes based on hydroponics–field rank correlation and stress tolerance indices

The evaluation of maize inbreds under WDS revealed significant differences in root architecture, physiological traits and grain yield (**Table 5**). Among the tested genotypes, ILM23 and ILM24 consistently exhibited superior performances across multiple traits. Both the lines possessed highly vigorous root systems (RT = 345 and 367; TL = 345 and 378; Seg = 415 and 787), robust physiological status (NDVI = 0.64 and 0.66; SPAD = 24.3 and 15.9), and the highest grain yields (3.56 and 3.22 kg/plot, respectively).

**Table 5:** Comparison of morpho-physiological and yield-related traits in tolerant maize lines under WDS condition.

Lines	PA	TL	RT	Forks	Seg	DiaA	ASI	NDVI	SPAD	KR	NKR	CL	CB	GY
Tolerant introgressed inbreds														
ILM23	298.5	345.6	345	226	415	3.46	0.00	0.64	24.3	16	18	15.5	30.15	3.56
ILM 24	456.8	378.4	367	344	787	1.91	1.00	0.66	15.9	18	26	6	44.6	3.22
Tolerant inbreds														
PML1249	176.3	289.2	218	235	215	1.00	2.00	0.59	23.8	12	21	6	35.01	2.56
PML1275	112.2	215.6	212	218	367	1.32	2.00	0.48	17.9	12	16	12	33.21	2.28
PML1285	235.8	368.8	209	279	401	0.99	3.00	0.47	25.4	10	18	12	36.16	2.21
LM22(check)	173.4	206.7	292	277	344	0.62	2.00	0.56	17.8	14	16	4.5	32.09	2.34
EML232	102.7	231.6	238	187	282	0.5	1.54	0.54	0.44	8	4	3.5	25.34	0.34

ASI, Anthesis silking interval (days); CB, Cob breadth(mm); CL, Cob length(cm); DA, Days to anthesis; DiaA, Average root diameter(mm); DiaM, Maximum root diameter(mm); EH, Ear height (cm); Forks, total number of forks; GY, Grain yield/plot(kg); KR, Total number of kernel rows; NDVI, Normalized difference vegetation index; NKR, Total Number of kernels/row; NL, Total number of leaves; NP, Number of plants; LL, Leaf length(cm); LW, Leaf width (cm); PA, Root projection area(cm^2^); PH, Plant height(cm); RT, Root tips(numbers); Seg, Total number of root segments; SPAD, Chlorophyll index; TL, Total Root Length.

These findings were further corroborated by the stress tolerance indices (**Table 6**). ILM23 and ILM24 recorded the highest STI values for RT (1.78 and 1.50, respectively) and TL (1.76 and 2.11, respectively), indicating strong resilience under stress, whereas their negative TOL values highlighted minimal yield reduction relative to optimal conditions. In contrast, tolerant inbreds, such as PML1285 and PML1275, displayed moderate STI and near-zero or slightly positive TOL values, whereas the other designated drought-tolerant line, LM 22 (Wajhat-un-nisa et al., 2023), recorded STI values (RT: 0.46; TL: 0.26), demonstrating the higher stress adaptation in the reported lines of this study. The susceptible lines either recorded no yield or very low yield, for example, EML 232 yielded 0.34 kg/plot with STI (RT: 0.21; TL: 0.18), and TOL (RT-0.64; TL -0.56).

**Table 6:** Comparative evaluation of maize inbreds using drought tolerance indices based on RT and TL.

Lines	RT_STI	RT_TOL	TL_STI	TL_TOL
Tolerant introgressed inbreds				
ILM23	1.78	-83	1.76	-45.51
ILM 24	1.50	-159	2.11	-50.9
Tolerant inbreds				
PML1285	1.91	-65	1.32	-0.14
PML1275	1.35	-45	0.79	-1.03
LM22(check)	0.46	-83	0.86	-29.82
EML232	0.21	-0.6	0.18	-0.56

RT, Root Tips; TL, Total Root Length; STI, Stress Tolerance Index; TOL, Tolerance Index.

Importantly, ILM23 and ILM24 were developed via marker-assisted breeding to introgress major WDS-tolerance QTL and belong to different heterotic groups derived from the parents of the previously released hybrid PMH 10. These introgressed lines, characterized by robust root systems, physiological resilience, and complementary genetic backgrounds, are ideal donor lines for developing WDS-tolerant maize hybrids. Collectively, these results provide a clear framework for selecting genotypes with strong root vigor and yield stability under water deficit stress for hybrid development.

**Figure 6:**
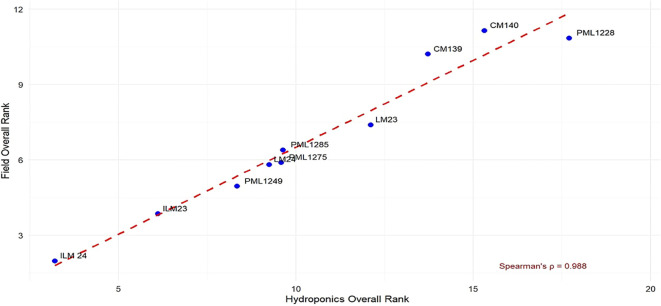
Correlation of genotype performance ranks between hydroponics and field environments. This scatter plot shows the field overall rank (y-axis) versus the hydroponics overall rank (x-axis) for the maize genotypes, demonstrating a strong positive correlation (Spearman's p=0.988) between the two testing environments. The strong positive correlation (Spearman's p=0.988) confirms that genotypes identified as superior in the controlled hydroponics system (ILM24, ILM23) maintained their high-performance ranking when tested under field condition.

**Figure 7:**
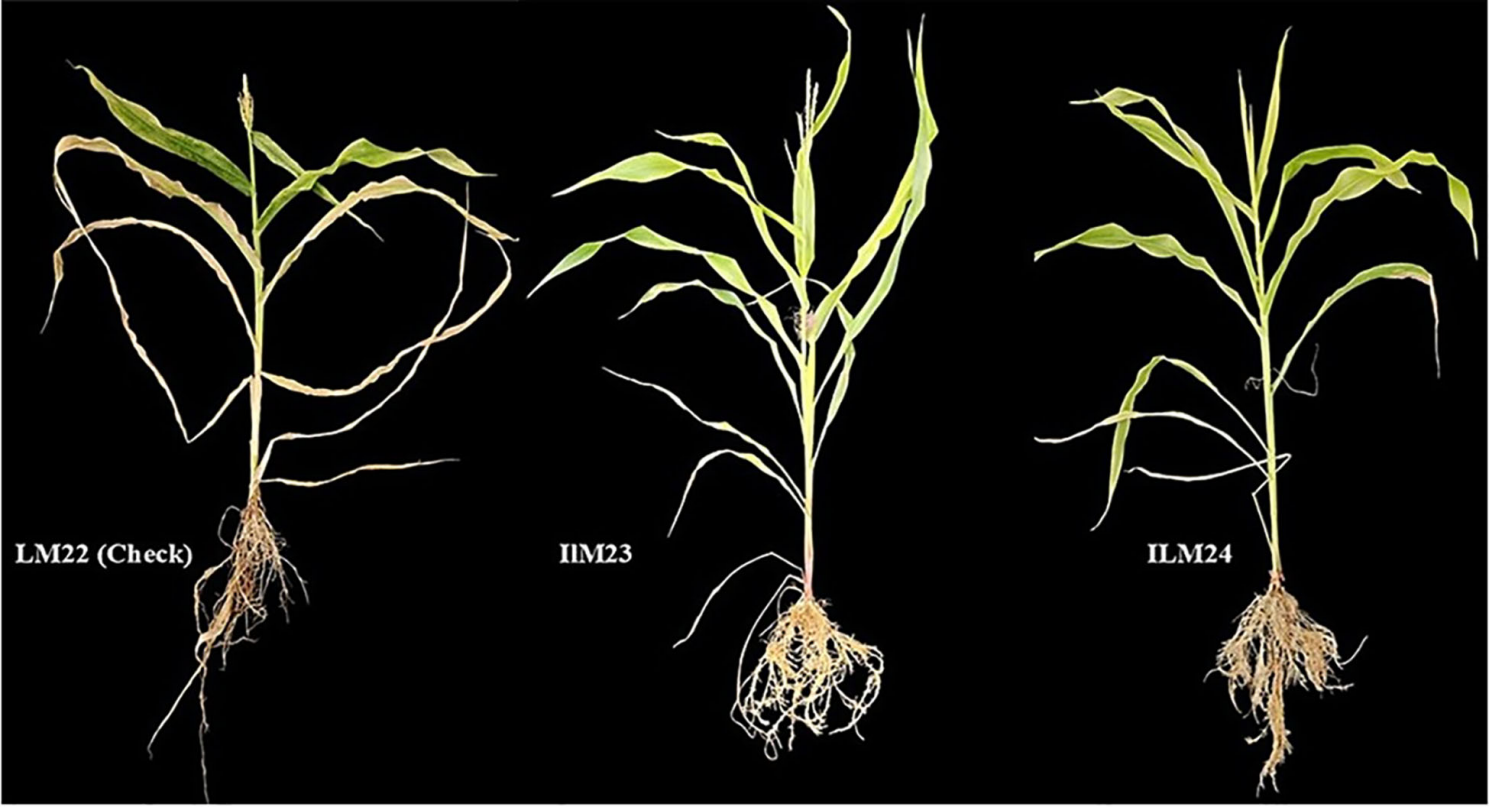
Visual assessment of root and shoot performance of maize inbreds under hydroponic screening at 60 DAS under WDS. The tolerant inbreds ILM23 (middle) and ILM24 (right) exhibit markedly improved root architecture and shoot vigour compared with the check LM22 (left), confirming their strong drought-adaptive traits under controlled water deficit conditions.

**Figure 8:**
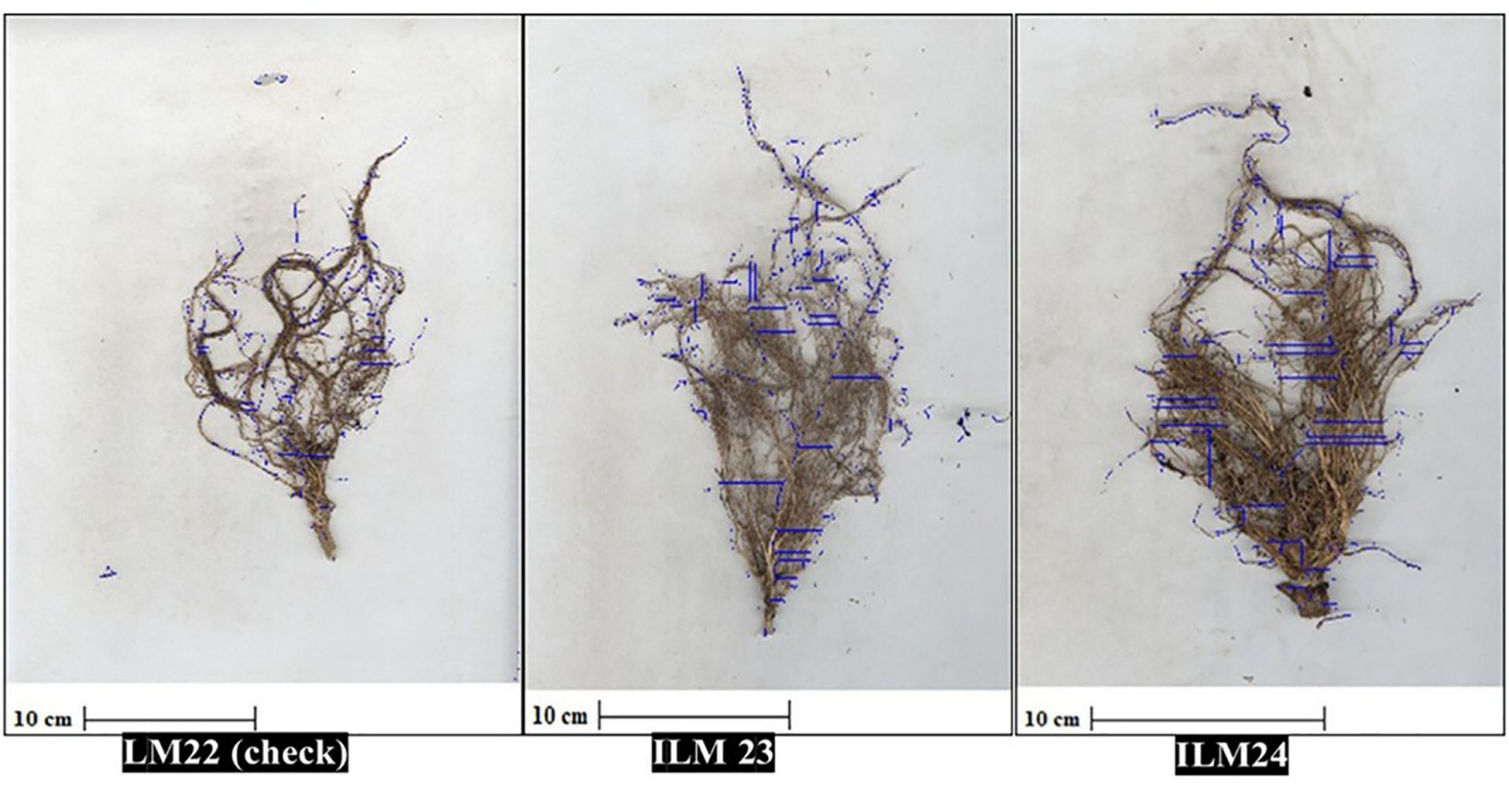
High-resolution root scans of maize inbreds at 60 DAS under hydroponic screening. Root imaging with the Biovis PSM R-2000 highlights pronounced differences between check (LM22) and tolerant (ILM23 and ILM24) lines under WDS, with the tolerant lines displaying more complex root architecture and increased root proliferation.

**Figure 9:**
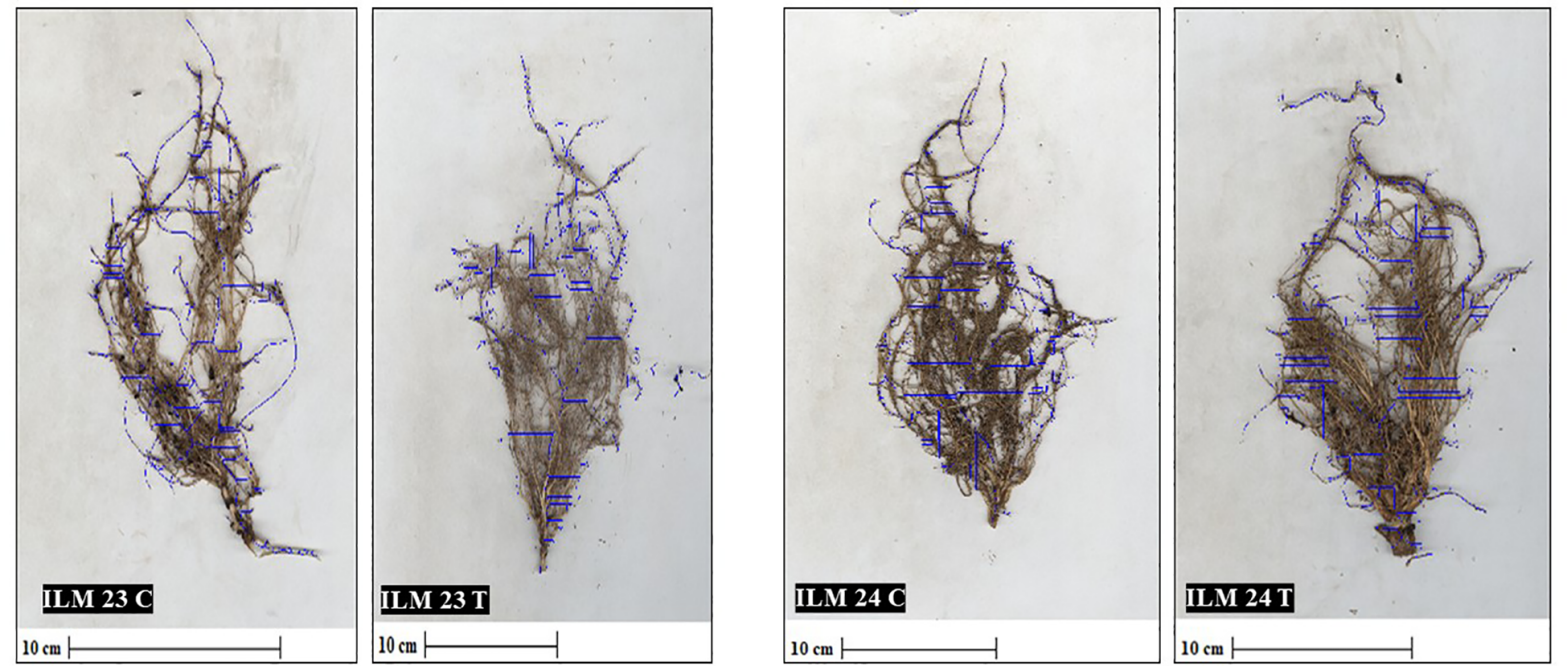
High-resolution root scans of maize introgressed lines at 60 DAS under hydroponic screening. Root imaging with the Biovis PSM R-2000 illustrates distinct differences between control (ILM23 C and ILM24 C) and WDS treated (ILM23T and ILM24T) plants. The WDS tolerant introgressed lines under treatment exhibit more complex root architecture and greater root proliferation compared with their respective controls.

Spearman’s rank correlation (ρ = 0.988) between hydroponics and field rankings demonstrated near-perfect concordance, confirming hydroponic screening as a reliable predictor of field performance under WDS (**Figure 6**). **Figure 7** illustrates the hydroponic screening of maize inbreds, where clear contrasts in shoot vigor and root development were evident. The tolerant lines, ILM23 and ILM24, outperformed the maize line, LM22, showing markedly stronger shoots and more elaborate root systems. High-resolution scans (**Figures 8**, **9**) reinforced these observations, capturing the pronounced root proliferation and architectural complexity that underpin WDS resilience.

## Discussion

WDS is a major abiotic constraint that limits maize productivity and affects cell division, elongation, photosynthesis and root anatomy (Hefny et al., 2017). Its impact is particularly severe during the vegetative and flowering stages, when reproductive tissues, root morphology and grain yield are most vulnerable (Chen et al., 2012; Sah et al., 2020; Shahzad et al., 2023). Therefore, screening germplasm for resilience is a key breeding priority. Historically, above-ground traits have been emphasized, whereas root traits have often been neglected due to destructive, labor-intensive phenotyping (Trachsel et al., 2010). A key contribution of this study is the optimization of a hydroponic protocol for early root phenotyping under PEG-induced osmotic stress, enabling the precise extraction and quantification of complex root traits. Osmolyte-driven polyethene glycol (PEG) is the best solute for imposing low water stress that is reflective of the type of stress imposed by drying soil (Kwasniewski et al., 2016), as these molecules generally do not enter the root and mimic the drought in a regulated manner. It lowers the external free water concentration without changing the ionic makeup of the cell and lowers leaf water potentials (Kumar et al., 2011). Hence, this low-volume hydroponic method offers high potential for dissecting the complexities in root architecture for WDS. While hydroponic systems do not entirely replicate the mechanical impedance, microbial interactions, and heterogeneous moisture gradients present in natural soils, they offer a highly controlled environment for isolating genotypic responses to water-deficit stress. The PEG-induced osmotic stress applied in this study effectively simulated drought conditions by lowering external water potential without altering ionic composition. Root phenotyping through the hydroponic system was further validated under field conditions following CIMMYT’s managed stress protocols. The near-perfect correlation between hydroponic and field rankings (ρ = 0.988) demonstrates that hydroponics can serve as a reliable, cost-effective first tier of selection, reducing breeding cycle time by identifying promising donors before field evaluations. Therefore, rather than replacing soil-based evaluation, the hydroponic platform can be best viewed as a rapid and reliable first-tier screening tool that enhances selection efficiency prior to field-level validation.

The combined results of ANOVA from root phenotyping and field evaluations provide strong evidence of genetic variability in maize root and yield-related traits under water deficit stress (WDS). The highly significant genotypic effects observed in both platforms underscore the presence of substantial genetic diversity, which can be exploited for developing drought-tolerant cultivars. The significant genotype × trait interactions further suggest that genotypes express differential adaptive strategies across traits, reflecting the complexity of drought response mechanisms. The absence of significant replication effects indicates high experimental precision and reliability of the results, supporting the robustness of the experimental design. Plasticity in root morphological traits such as rooting depth, root diameter, the number of root branches, length of root hairs and projection area determines the adaptation for improved water use efficiency in crop species (Sah et al., 2020). Boxplot analyses revealed that traits such as DiaM, Forks and Seg were reduced under WDS. This reduction likely reflects an energy-conserving strategy, where plants limit carbon allocation to lateral branching and thicker roots (Comas et al., 2013). Conversely, the increase in RT and TRL highlights a compensatory mechanism aimed at enhancing soil exploration under limited water availability (Kou et al., 2022; Ashley et al., 2024). It is well documented that WDS stress promotes preferential assimilate partitioning toward the root system, resulting in an increased root:shoot ratio that enhances water capture from deeper or less-depleted zones (Ali et al., 2016). The enhanced total root length and increased number of root tips observed in tolerant inbreds in this study also reflect such strategic carbon allocation, prioritizing root elongation over lateral expansion as an energy-efficient mechanism under stress (Jain et al., 2019). Moderate reductions in NDVI and projection area suggest that both shoot vigor and root expansion are partially constrained under stress, though the presence of significant genotypic variation highlights potential for selecting superior performers under WDS (Keerthi et al., 2025; Kaur et al., 2025). To further explore the intricate relationship between various root traits and yield attributing traits, correlation analysis was carried out. Root traits, such as tips, TRL, forks and seg emerged as the most critical determinants of yield stability, showing strong correlations (r ≥ 0.70) with kernel traits, NDVI, and grain yield. This plasticity, which involves reduced lateral branching but elongation of fine roots under stress, is consistent with adaptive strategies that enhance soil exploration while conserving energy (Kou et al., 2022). A more extensive root system enables continued water and nutrient uptake during critical reproductive phases, thereby protecting processes such as pollen viability, silk emergence and kernel filling (Jiang and Whalen, 2025). In maize, maintenance of plant water status during flowering is directly linked to reduced anthesis–silking interval (ASI), improved kernel set and higher final grain yield (Sah et al., 2020). Multivariate analysis further highlighted the synergy between root vigor, canopy greenness, and reproductive efficiency, aligning below-ground resilience with above-ground stability.

Donor identification is another major outcome of this study. Among the 50 inbreds evaluated, ILM23 and ILM24 consistently emerged as core donor resources, combining vigorous root systems (high RT, TL, and Seg) with a maintained ASI and superior grain yield under stress. PML1249, PML1275, and PML1285 complemented these as secondary but valuable donor sources, broadening the reservoir of adaptive traits. Together, these five genotypes form a robust donor panel for WDS resilience, offering immediate value for breeding programs targeting high water-use efficiency (WUE) and stress-tolerant maize hybrids. In the present study, genotypes exhibiting greater root length and higher root tip also maintained relatively stable NDVI values under stress, indicating prolonged canopy greenness and sustained photosynthetic activity. This, in turn, ensures a continuous supply of assimilates to developing kernels, mitigating the negative effects of drought on grain filling. The combined root and shoot resilience thus provide a mechanistic explanation for the superior yield performance of tolerant inbreds such as ILM23 and ILM24 under WDS. The genetic pedigree of ILM23 and ILM24, developed through marker-assisted introgression of major WDS-QTL into distinct heterotic groups (Kaur et al., 2025), further strengthens their utility. Their complementary backgrounds and favorable stress tolerance indices highlight their strategic roles in future hybrid development.

In summary, this study contributes to three major advances.

1. A two-tier phenotyping platform that integrates hydroponics and field evaluation, combining efficiency and predictive reliability.
2. An optimized hydroponic protocol enabling precise and scalable root phenotyping under WDS.
3. Identification of a donor panel, led by ILM23 and ILM24 and supported by PML1249, PML1275, and PML1285, that provides immediate resources for breeding resilient maize.

These findings reinforce the concept that root traits are not merely adaptive survival traits, but key contributors to yield stability, making them valuable indirect selection targets in WDS-resilient maize breeding. Integrating root traits into early selection can accelerate the development of cultivars with stable productivity under water-limited environments, bridging the gap between controlled screenings and field performances.

## Conclusion

This study demonstrates that integrating hydroponic and field platforms provides a precise and efficient framework for screening maize under water-deficit stress. The strong concordance between root traits and field performance highlights hydroponic phenotyping as a reliable first-tier tool to accelerate the breeding of resilient, high-yielding cultivars.

## Data Availability

The original contributions presented in the study are included in the article/supplementary material. Further inquiries can be directed to the corresponding author.
